# Retraction: FSM-F: Finite State Machine Based Framework for Denial of Service and Intrusion Detection in MANET

**DOI:** 10.1371/journal.pone.0343279

**Published:** 2026-02-23

**Authors:** The PLOS One Editors

The *PLOS One* Editors retract this article [1] due to concerns about:

- The use of terminology that differs from standards in the field (e.g. “interruption discovery” instead of “intrusion detection”),
- Text in section “Packet Dropping Attack” that appears similar in content to a previously published article [2] that was not cited.
- Text in section “Interruption Detection AODV (ID-AODV)” that appears similar in content to a previously published article [3] that was cited as Reference 20.

All authors either did not respond directly or could not be reached.
